# Visualization of Heart Rate Variability of Long-Term Heart Transplant Patient by Transition Networks: A Case Report

**DOI:** 10.3389/fphys.2016.00079

**Published:** 2016-03-07

**Authors:** Joanna Wdowczyk, Danuta Makowiec, Karolina Dorniak, Marcin Gruchała

**Affiliations:** ^1^1st Chair and Clinic of Cardiology, Medical University of GdańskGdańsk, Poland; ^2^Institute of Theoretical Physics and Astrophysics, University of GdańskGdańsk, Poland; ^3^Department of Noninvasive Cardiac Diagnostics, 2nd Chair of Cardiology, Medical University of GdańskGdańsk, Poland

**Keywords:** heart transplantation, heart rate variability, cardiac autonomic modulation, reinnervation, arrhythmias, hearth rhythm dynamics

## Abstract

We present a heart transplant patient at his 17th year of uncomplicated follow-up. Within a frame of routine check out several tests were performed. With such a long and uneventful follow-up some degree of graft reinnervation could be anticipated. However, the patient's electrocardiogram and exercise parameters seemed largely inconclusive in this regard. The exercise heart rate dynamics were suggestive of only mild, if any parasympathetic reinnervation of the graft with persisting sympathetic activation. On the other hand, traditional heart rate variability (HRV) indices were inadequately high, due to erratic rhythm resulting from interference of the persisting recipient sinus node or non-conducted atrial parasystole. New tools, originated from network representation of time series, by visualization short-term dynamical patterns, provided a method to discern HRV increase due to reinnervation from other reasons.

## 1. Introduction

In patients with end-stage heart disease, heart transplantation (HTx) is associated with significant improvement in survival and in quality of life. However, after HTx multiple adverse events may occur, including acute rejections, graft failure, infections, allograft vasculopathy and arrhythmias including atrial fibrillation, all of which adversely affect long term survival. Complete denervation of the transplant significantly affects reactivity of transplanted heart and hemodynamics of circulation (Willmann et al., 1963).

Cardiac reinnervation, both sinus and myocardial, was extensively studied with a variety of methods, including heart rate variability (HRV), PET with C-11 hydroxyephedrine, MIBI-SPECT and others, and proved non-uniform and occurring at variable time (Kaye et al., 1993; Uberfuhr et al., 2000; Vanderlaan et al., 2012; Imamura et al., 2014). Higher HRV indices were associated with graft reinnervation by many authors (Uberfuhr et al., 2000; Cornelissen et al., 2012; Vanderlaan et al., 2012). On the other hand, low HRV values were associated with hypertension and increased incidence of graft vasculopathy, even in pediatric patients (Giordano et al., 2013). Moreover, HRV values in HTx patients can be confounded by certain transplant-specific factors, such as type of the surgery and time passed after the surgery (Uberfuhr et al., 2000). Nevertheless, HRV analysis is commonly assumed as providing insight in both sympathetic and parasympathetic allograft sinus reinnervation, which was demonstrated to improve outcome (Vanderlaan et al., 2012).

We present a HTx patient at his 17th year of uncomplicated follow-up. Within a frame of routine check out several tests were performed. With such a long and uneventful follow-up some degree of graft reinnervation could be anticipated. However, the patient's ECG Holter and exercise parameters seemed largely inconclusive in this regard. The exercise heart rate (HR) dynamics were suggestive of only mild, if any parasympathetic reinnervation of the graft with persisting sympathetic activation via plasma catecholamines. On the other hand, traditional HRV indices were inadequately high, due to erratic rhythm resulting from interference of the persisting recipient sinus node or non-conducted atrial parasystole.

To elucidate post-HTx HRV values (e.g., to discern between HRV increase due to post-HTx rhythm alterations such as non-conducted atrial parasystole, recipient sinus activity or true reinnervation effect), new tools are clearly needed. Here we represent the short-term dynamics of nocturnal RR-intervals by the novel complex network tools (a transition network and its adjacency matrix, Donner et al., 2010; Makowiec et al., 2014, 2015) and explore the visualization of this representation in assessment of HRV.

## 2. Background

### 2.1. Case presentation

A 68-year-old male patient 17 years after heart transplant was referred to our outpatient clinic for a regular follow-up. The patient was transplanted by biatrial method due to end-stage heart failure following a viral infection in July 1997. The Donor was a 37-year-old male without known risk factors. Postoperative course was uneventful under immunosuppression therapy with cyclosporin with no rejection episodes. The patient's post-HTx history included well controlled hypertension, mild chronic kidney disease, and prostatic hypertrophy.

Physical examination revealed good overall clinical condition, with normal blood pressure of 140/90 and a regular heart rate of 85/min. Standard laboratory findings were within reference ranges except for creatinine level of 1.5 mg/dL and GFR of 45 mL/min. Standard ECG demonstrated a regular sinus rhythm of 85 bpm, QRS duration within normal range and no ST segment deviation or T-wave abnormalities. Echocardiography revealed normal left ventricular function with EF of 60% and mild left ventricular hypertrophy of the graft that was noted during follow-up. Coronary angiography (2 years prior) showed normal coronary arteries without features of graft vasculopathy. Last myocardial biopsy performed in 2013 showed no signs of cellular rejection (ACR Grade 0 R). Standard graded cycle ergometry exercise test limited by exhaustion was performed (target HR was not used). Resting heart rate (82 beats/min) and BP values (140/84 mmHg), slowly increased up to 100 beats/min and 185/90 mmHg, respectively over 7 min. Maximal workload achieved was 75 W. During recovery phase a delayed HR recovery was noted, with HR gradually returning to its baseline value over 9 min.

### 2.2. HRV analysis

**Figure 1:**
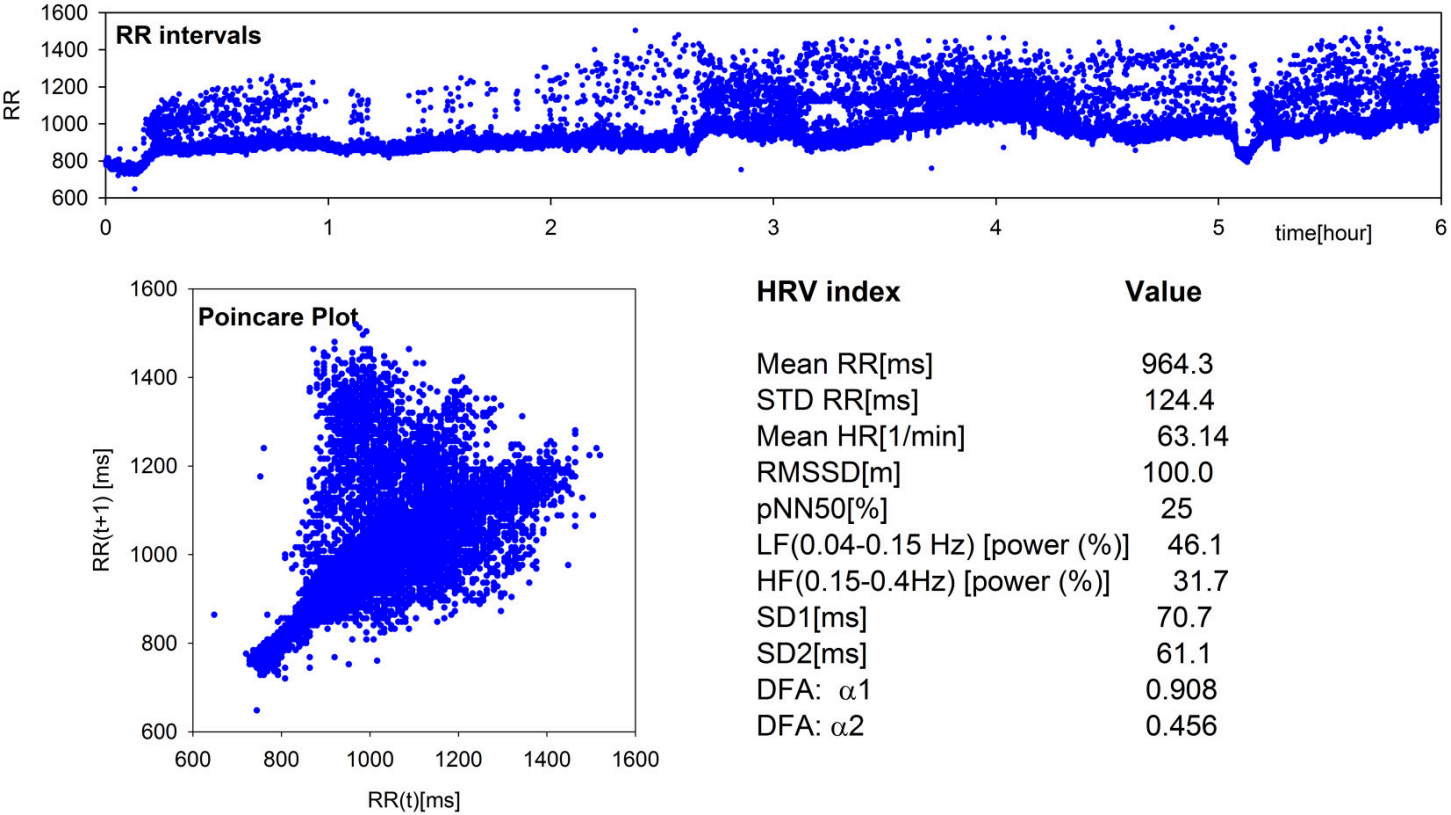
**Elements of standard and non-linear HRV analysis: plot of RR-intervals time series, Poincare Plot, and values of the main HRV indices obtained from the 6-h nocturnal RR-signal of the patient under study**. Estimates were performed by Kubios HRV Pro V2.0 (Tarvainen et al., 2014).

24-h Holter monitoring was performed. The recording was first analyzed using Del Mar Impresario Software. Time domain and frequency domain analysis of HRV was carried out, preceded by visual inspection of the automatic ECG recording. All supraventricular and ventricular extrasystoles were excluded. The nocturnal 6-h part was determined following the evident day-night switch in the length of RR-intervals. Highly irregular sinus rhythm pattern erratic rhythm - together with values of basic indices of HRV can be seen in Figure 1 (by Kubios HRV Pro V 2.0, Tarvainen et al., 2014). Close ECG examination revealed non-conducted atrial parasystole and P wave originated from the recipient sinus rhythm resulting in inappropriately high values of time and frequency measures. Non-linear analysis methods were also used. Long-term fractal scaling exponent measured by the detrended fluctuation analysis method in long-term RR-interval fluctuations and the shape of Poincare plot of each RR-interval vs the next, demonstrated increased randomness of heart rate patterns.

**Figure 2:**
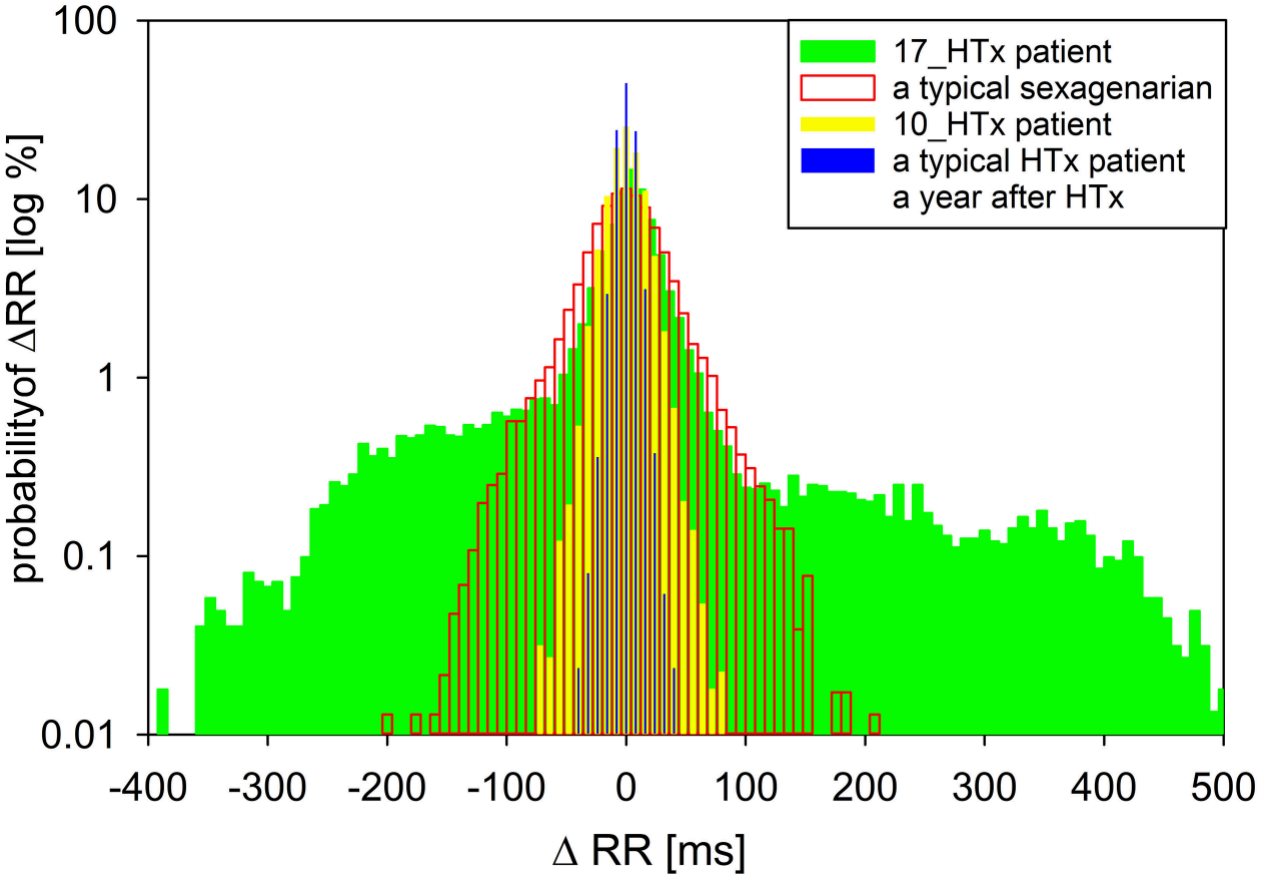
**A comparison of the distribution of increments Δ*RR*(*t*) = *RR*(*t*) − *RR*(*t* − 1) for the nocturnal 6-h signal obtained from the patient under study when he was 17 years after HTx to distributions of RR-increments obtained from nocturnal recordings of a typical sexagenarian, the patient under study when he was 10 years after HTx and a typical HTx patient when a person was a year after HTx**.

For additional analysis, a transition network—a novel network tool of time series representation (Donner et al., 2010; Makowiec et al., 2014, 2015), was applied to changes in subsequent RR-intervals, called RR-increments. The transition network is constructed from ordered RR-increments (called vertices) which are connected by an edge if two RR-increments are adjacent in time. Repetitive edges are represented by a weight of an edge. In consequence, each vertex has the weight (a total of weights of adjacent edges) which means probability of a given RR-increment. In Figure 2, the probabilities of RR-increments are shown. Additionally, there are given probabilities obtained for a healthy typical coeval, a typical HTX patient early after surgery, and the same patient but 7 years earlier. The noticeable over-presence of large accelerations (larger than 100 ms) and large deceleration (larger than 100ms) in the signal of the considered patient explains huge values of standard HRV indices.

**Figure 3:**
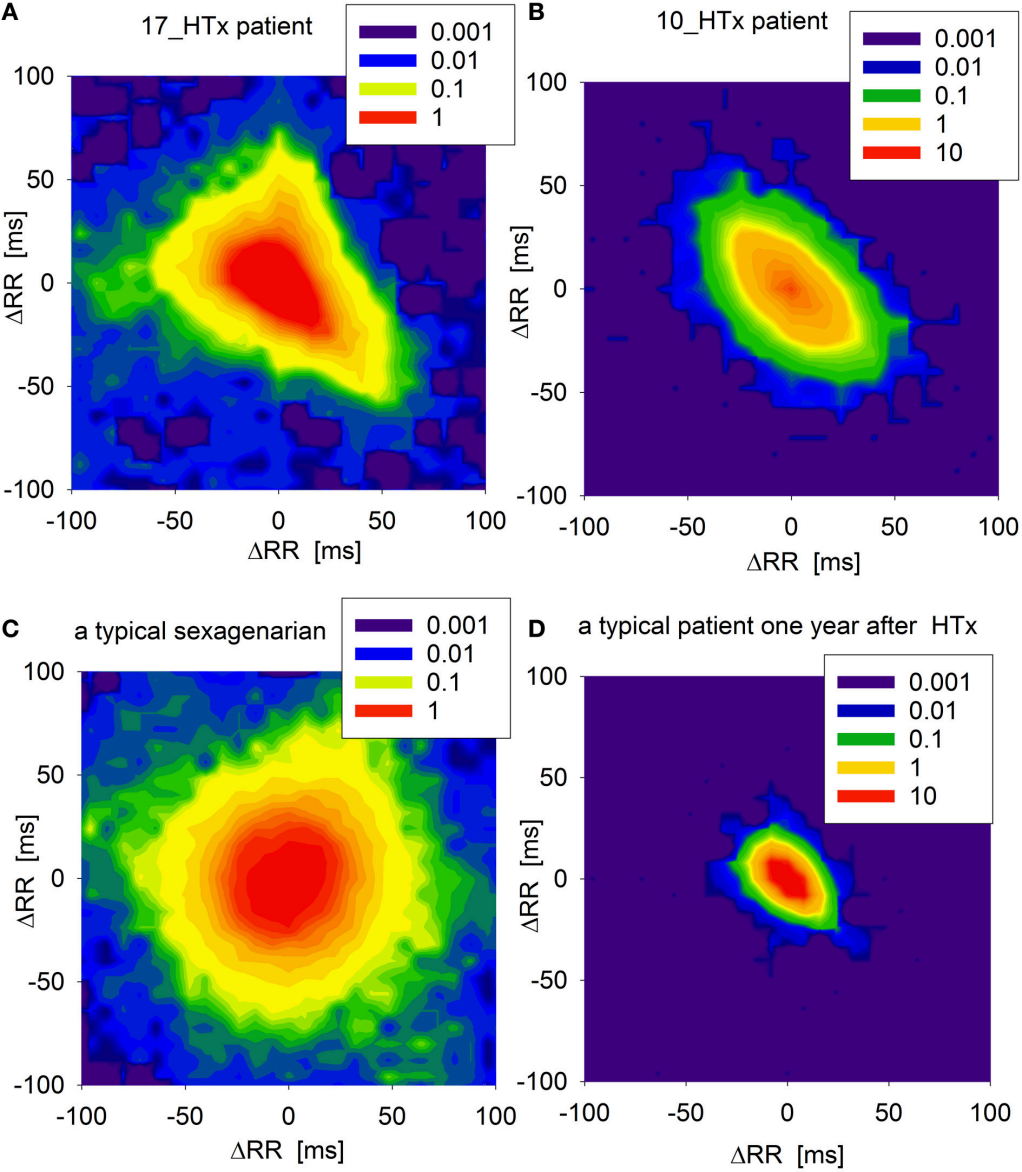
**Adjacency matrices of transition networks obtained from increments Δ*RR* as probability density plots (in log[%]) of the core parts of networks, (i.e., when accelerations and decelerations Δ*RR* are smaller than 100 ms) for: (A) the patient under study when he was 17 years after HTx, (B) the patient under study when he was 10 years after HTX, (C) a typical sexagenarian, and (D) a typical HTx patient a year after HTx**.

An adjacency matrix is a mathematical representation of weights of edges in a transition network. It shows probability that a pair of RR-increments appears consecutively in a time series. In Figure 3, the adjacency matrices are shown as density plots of the same signals as in Figure 2. These plots present the probabilities of physiologically justified accelerations and decelerations (RR-increments smaller than 100 ms). The resulting density plot organization in the patient at 17th year post-HTx is markedly different from the respective plots in a recently transplanted HTx patient (1 year post-HTx) or in a healthy sexagenarian volunteer. It is also clearly different from previous examinations of our patient (conducted at the 10th year post-HTx).

## 3. Discussion

Following HTx, the loss of autonomic input to the allograft, (with greater impact of reduced parasympathetic activity), results in persistently elevated resting heart rate and decreased chronotropic reserve (Willmann et al., 1963). This is illustrated by the highly condensed adjacency matrix in a patient 1 year post-transplant (see, Figure 3). Decreased HRV indices at various time points of the post-transplant period suggest that the transplanted heart may have not be reinnervated. However, a degree of vagal reinnervation as assessed by HF power spectral analysis was demonstrated as early as in the first 6 months post-HTx (Imamura et al., 2014). Sympathetic reinnervation (as assessed by increased LF/HF and total power) has also been documented over the first 18 months post-HTx, though it is not simply a function of time. Reinnervation was shown to be more likely at younger age, after short uncomplicated surgery, and with low rejection rates (Bengel et al., 2002). Moreover, HRV indices can potentially be misleading due to interfering recipient sinus activity or non-conducted arrhythmias in some patients. Nevertheless, HRV analysis was used extensively to assess autonomic reinnervation in HTx recipients as it still is the most accessible clinical surrogate of reinnervation (Bengel et al., 2001; Imamura et al., 2014).

In our patient, HR response to workload during the first minutes of exercise was restored to some extent, not only due to some degree of reinnervation but also it continues to respond to plasma catecholamines (Kaye et al., 1993). Also, his baseline HR was close to 80 bpm, which is similar to resting HR reported by Imamura in patients with presumed post-HTx reinnervation (Imamura et al., 2014) as opposed to those lacking innervation in whom resting HR was at the level of 90 bpm (Wilson et al., 2000). HR recovery, however, was suggestive of poor parasympathetic reinnervation in our patient (Imamura et al., 2015).

When the biatrial method of heart transplantation is used, several specific heart rhythm alterations may occur. Activation of the recipient atrial tissue may be evident on ECG. In combination with graft P waves the native P waves may mimic atrial flutter (Elsik et al., 2012). Reestablishment of conduction across atrial anastomosis may produce recipient-to-donor conduction of sinus beats, atrial parasystole or tachycardia because of fibrillatory activity in the recipient atrium. Sinus activity of the recipient atrium may also escape into the donor atrium intermittently. Thus, in addition to reinnervation, an increasingly conduction of the suture line between the recipient atrium and the donor atrium could produce atrio-atrial on/off mechanism, predisposing to erratic rhythm and falsely increased HRV. The scars in the atria act as conduction barriers and can also predispose to atrial flutter (Elsik et al., 2012). In this case part of the heart rate variability (HRV) arises from a biatrial surgical technique and interference of the persisting recipient sinus node, see Figure 2. This situation makes it questionable whether any type of analysis of the HRV allows conclusions about the degree of reinnervation.

Sleep, in general, can be assumed as a period of human activity which is free of external stimulation. Therefore, the nocturnal part of a 24-h Holter recording provides a good possibility of observing the state of the autonomic baseline (Stein and Pu, 2012; Chouchou and Desseilles, 2014; Makowiec et al., 2015). A recent review (Stein and Pu, 2012) advocates using nocturnal records for HRV analysis. However, as sleep is organized in cycles, switches between non-REM (with high vagal activity) and REM (increased sympathetic stimulation) sleep may contribute to the observed erratic patterns.

As shown in Figure 3, signals from a healthy volunteer result in transition networks with many dynamics patterns playing an equivalent role. Signals from a typical HTx patient in the early post-transplant period provide networks in which accelerations are more probable to be followed by decelerations and vice verse. Moreover, the transitions are concentrated around the smallest RR-increments possible. The similar structure, but extended to RR-increments of larger size, can be read from the plot of the patient 10 years after HTx. Such a picture can be seen as the increase of direct autonomic regulation resulting from the emergence of reinnervation. However, the signal from our patient recorded 17 years post-HTx shows strange asymmetry between accelerations and decelerations suggesting that the basic dynamical pattern is distinct from both a healthy sexagenarian and a healthy post-HTx patient.

## 4. Concluding remarks

We have presented a case of a patient, many years after heart transplant, with good functional status, and discussed if dynamics of RR-intervals and RR-increments as assessed by tools based on complex network analysis applied to 24-h ECG recording, could provide more insight into physiological background of the standard HRV measures. Especially we expected to find means for assessing the reinnervation process. However, we have found a strong presence of erratic rhythms which dominated the classic HRV information and gave the false description about the activity of autonomic system. Nevertheless, this finding is important for the patient as it could switch further to supraventricular arrhythmia.

The method used—graphs of adjacency matrices of transition networks, can be compared to the technique of Poincare plots, now applied to signals of RR-increments. By its very nature it offers insight into dynamical interbeat dependencies: are they stochastic like (rhythm of sexagenarians) or fixed (a year after HTx) or structured irregular (with erratic rhythms). Obviously, our analysis can be extrapolated to larger group of subjects, especially, in a follow-up study because development of arrhythmias increases with time passed after the HTx (Thajudeen et al., 2012). We work on a full paper regarding validity of the method in which we compare groups of patients at different stages after HTx.

Further research based on augmented pool of data is needed, to assess clinical applicability of the new tools and their potential to provide additional insight in graft electrophysiology beyond standard surrogate measures of reinnervation, such as classical HRV analysis and HR response to workload. High vigilance and advanced diagnostic tools to assess allograft status are mandatory to allow for appropriate intervention in allograft-related syndromes that may emerge with improved survival of patients after transplantation.

## Informed consent statement

The study complied with the Declaration of Helsinki and was approved by the Bioethics Commission for Research of the Medical University of Gdańsk. The written informed consent was obtained from the study subject.

## Author contributions

JW, DM, and MG: substantial contributions to the conception and design of the work; JW: the acquisition of the data; KD: interpretation of data, revising it critically for important intellectual conent; MG: final approval of the version to be published; JW, DM: analysis, and interpretation of data, drafting the work, revising it critically for important intellectual content and approval of the final version to be submitted; the final revision of the resubmission. All authors agreed to be accountable for all aspects of the work in ensuring that questions related to the accuracy or integrity of any part of the work are appropriately investigated and resolved.

### Conflict of interest statement

The authors declare that the research was conducted in the absence of any commercial or financial relationships that could be construed as a potential conflict of interest.
